# Adaptive Intuitionistic Fuzzy Enhancement of Brain Tumor MR Images

**DOI:** 10.1038/srep35760

**Published:** 2016-10-27

**Authors:** He Deng, Wankai Deng, Xianping Sun, Chaohui Ye, Xin Zhou

**Affiliations:** 1 Key Laboratory of Magnetic Resonance in Biological Systems, State Key Laboratory of Magnetic Resonance and Atomic and Molecular Physics, National Center for Magnetic Resonance in Wuhan, Wuhan Institute of Physics and Mathematics, Chinese Academy of Sciences, Wuhan 430071, China; 2 Department of Head and Neck and Neurosurgery, Hubei Cancer Hospital, Wuhan 430079, China

## Abstract

Image enhancement techniques are able to improve the contrast and visual quality of magnetic resonance (MR) images. However, conventional methods cannot make up some deficiencies encountered by respective brain tumor MR imaging modes. In this paper, we propose an adaptive intuitionistic fuzzy sets-based scheme, called as AIFE, which takes information provided from different MR acquisitions and tries to enhance the normal and abnormal structural regions of the brain while displaying the enhanced results as a single image. The AIFE scheme firstly separates an input image into several sub images, then divides each sub image into object and background areas. After that, different novel fuzzification, hyperbolization and defuzzification operations are implemented on each object/background area, and finally an enhanced result is achieved via nonlinear fusion operators. The fuzzy implementations can be processed in parallel. Real data experiments demonstrate that the AIFE scheme is not only effectively useful to have information from images acquired with different MR sequences fused in a single image, but also has better enhancement performance when compared to conventional baseline algorithms. This indicates that the proposed AIFE scheme has potential for improving the detection and diagnosis of brain tumors.

Brain tumor is one of the most fatal cancers, especially high-grade gliomas are amongst the most deadly^1^. National Cancer Institute estimates that the number of new cases of brain and other nervous system cancers was 6.4 per 100,000 people per year, and the number of deaths was 4.3 per 100,000 people per year^2^. However, there are no valid ways to prevent brain tumors^3^. At present, treatments of brain tumors in early stages are successful and effective. Thus, early detection and diagnosis of brain tumors are crucial to decrease the mortality. Several principal imaging techniques are utilized for brain examination, including computerized tomography (CT), positron emission tomography (PET), magnetic resonance (MR) imaging, and ultrasound imaging. Among them, MRI is the most common technique for radiologists and surgeons to detect and diagnose brain tumors^4^,^5^,^6^, because it can non-invasively provide images with good soft-tissue contrast and high spatial resolution.

Typically multiple MR sequences are utilized to image different interesting structures for diagnosis and delineation of brain diseases^7^, for example, T1-weighted, T2-weighted, contrast-enhanced T1-weighted and T2-FLAIR (T2-weighted with fluid-attenuated inversion recovery) sequences. Each sequence provides diverse information, as shown in Fig. 1 (One axial slice of a male patient with diffuse astrocytoma, age of 30, scanned by Eclipse 1.5 Tesla in Hubei Cancer Hospital). T1-weighted images are known as ‘anatomy scans’, and they are usually used for the structural analysis. As shown in Fig. 1(a), the tumor and focal edema appear dark, whose location is indicated by an arrow. The normal tissues are easily annotated, such as the sulcus, callosum and cerebrospinal fluid (CSF). T2-weighted images are known as ‘pathology scans’ because abnormal regions (e.g., the edema and tumor) and the CSF are bright against the dark normal brain tissues, as shown in Fig. 1(b). The arrow indicates the location of abnormal regions. In order to separate the tumor from surrounding focal edema, a contrast-enhanced T1-weighted image (gadolinium-DTPA) is necessary, as shown in Fig. 1(c), where the arrow denotes the location of the tumor. The tumor borders appear bright because the contrast agent accumulates there due to the disruption of blood-brain barrier in the proliferative tumor region^5^. Figure 1(d) suggests that the focal edema is easily separated from the CSF because the free water signal is suppressed in a T2-FLAIR image. The arrow denotes the location of the focal edema.

When a radiologist/surgeon views brain tumor MR images, he or she usually compares the images from one imaging modality to other ones, and then observes morphological abnormalities as well as describes their locations repeatedly. This is a time-consuming process, which may fail to identify some subtle findings in MR images. Meanwhile, low-grade tumors (especially the first stage gliomas) are generally represented by T1 and T2 sequences very similar to healthy brain tissues. This arouses great difficulties in diagnoses.

Moreover, due to limitations of MR hardware systems, brain MR images are undoubtedly degraded by various types of noise, or blurred owing to the physical properties of imaging devices. This may present poor contrast or low resolution, hindering the discrimination and diagnosis of brain tumors^5^. Accordingly, many methods are utilized to improve the visual quality of brain MR images. One method is to collect more images at the data acquisition stage, which augments the overall acquisition time, radiologist’s caseload, and hardware costs. The other way is to ameliorate the contrast of specific and/or interesting regions in images during the post-processing stage without influencing the acquisition process or burdening the hardware costs.

Nonlinear filtering methods (such as the median or weighted median filtering^8^, maximization of information content^9^, or algorithms based on brightness selection and edge detection^10^) can effectively preserve edges, details, or intensities of the image. Morphology methods are useful in representation and description of region shape (e.g., skeletons, boundaries, and convex hull)^11^. Histogram equalization-based methods hold the dominant position in the field of enhancement^6^,^12^. However, inherent incapacities for the preservation of brightness and the washed out effect block the development of those methods^13^. Owing to the unavoidable ambiguity and uncertainty during the image acquisition and transmission, as well as the fuzziness of object description in medical images, fuzzy sets (FSs)-based methods have been presented to improve the image contrast in recent years^14^,^15^,^16^,^17^. But those methods may produce artifacts, or not well enhance all local details/regions. Furthermore, the above pay little attention to the synchronous presentation of diverse filtered results under different imaging modalities in an image, though this is beneficial for radiologists/surgeons to understand normal and abnormal structures of the brain. Therefore, the enhancement of brain tumor MR image is still an open issue.

In order to design an efficient enhancement method, it is inspiring to adopt intuitionistic fuzzy sets (IFSs) because IFSs consider more uncertainties in comparison to classical FSs. In recent years, IFSs have drawn much attention in image processing^17^,^18^,^19^,^20^. Among these methods, the theoretical properties of IFSs model are investigated from mathematical viewpoints. This is helpful to guide the design of new enhancement way. Notice that fusion operators can integrate the highlighted fine details under diverse imaging modes with original ones, and display the results in a single image. With these considerations in mind, we design a new enhancement method of brain tumor MR images inspired by the IFSs model and fusion operators in this paper.

The contributions of this paper can be summarized as follows: i) An efficient fuzzy filtering scheme inspired by the IFSs model is proposed. This scheme successively implements different intuitionistic fuzzification, hyperbolization and defuzzification operations on each object/background area of an input MR image. These implementations can be parallel processed. The proposed fuzzy filtering scheme can improve the contrast and visual quality of the images under different imaging modalities. ii) A nonlinear fusion scheme is proposed to synchronously display the improved normal/abnormal structures of the brain as a single image. This can be advantageous for radiologists/surgeons to understand abnormalities appearing in the brain, and improve the disease diagnosis. These recently proposed techniques simultaneously improve the contrasts of brain fold, deep brain structure (e.g., brain nuclei), corpus callosum, focal edema, and/or active tumor ingredients. We use real patient data to test the proposed method. Experimental results demonstrate that the proposed method is not only efficient with respect to the improvement of normal/abnormal regions of a brain, but also has better performance when compared with conventional baseline methods.

## Methods

### Theory

Integrating intuitionistic fuzzy filtering (called as NLFI) with fusion operators, we propose a new enhancement scheme, denoted as AIFE, to enhance brain tumor MR images. Assume that *U* is a space of points set, a fuzzy set *A* = {(*u*, *μ*_*A*_(*u*),*ν*_*A*_(*u*))|*u*∈*U*} is represented by a membership function *μ*_*A*_(*u*), where *u* denotes a generic element of *U*. In this case, *μ*_*A*_(*u*) and *v*_*A*_(*u*) are the membership and non-membership degrees. The *μ*_*A*_(*u*) associates a real number within an interval [0,1] with each point in *U*.

Compared with classical FSs, IFSs reflect better the aspects of human behaviors. Besides the presence of vagueness, IFSs offer a solid, flexible and mathematical setting to deal with the hesitancy derived from imperfect/imprecise data^17^,^21^. For a finite *U*, its intuitionistic fuzzy set *A* is,


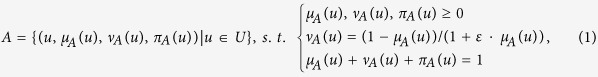


where *ε* is a constant within an interval [0,1], and *μ*_*A*_(*u*), *ν*_*A*_(*u*) and *π*_*A*_(*u*) denote the membership degree, non-membership degree and hesitation degree, respectively.

An image could be defined as an array of fuzzy singletons indicating the membership value of each pixel point. The *μ*_*A*_(*u*) in Equation (1) can be constructed by *S* and *Z* functions, logarithmic functions, exponential functions, and restricted equivalence functions (REFs), and so on^22^. We utilize REFs to construct the membership function in this paper.

A REF can be defined as the following formula.


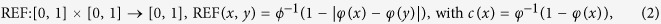


where *φ* and *ϕ* are two automorphisms in the unit interval, and *c*(*x*) is a strong negation. Let *φ*(*x*) = log((*e* − 1)*x* + 1) and *ϕ*(*x*) = *x*^2^, thus a REF is


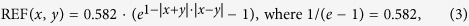


It is easy to prove that Equation (3) satisfies the conditions of REFs. Different to Equation (3), a Chaira’s REF was cREF(*x*,*y*) = 0.582∙(*e*^1−|*x*−*y*|^−1)^17^. However, Chaira’s REF might produce some problems in particular applications, such as segmentation or classification. The reasons are analyzed as follows. A local area of size 9 × 9 with 81 gray levels and its gray map are shown in Fig. 2. The average gray value of this area is 144 (viz., blue rectangular box). We decompose the area into part *A* and part *B*, where the blue line is the separatrix. When *x* denotes the gray value at pixel point of part *A* or part *B*, and *y* relates to the average value of that part, the intuitionistic fuzzy divergence^17^ between part *A* and part *B* is zero according to Chaira’s REF (via normalization). In this way, part *A* is so much like part *B* in the membership plane, which is inconsistent with the original gray difference. However, according to Equation (3), the divergence between part *A* and part *B* is 0.3519 rather than 0 (the parameter in the intuitionistic fuzzy divergence was set to 0.6). This indicates that the REF based on Equation (3) is suitable to discriminate part *A* and part *B*.

Therefore, we substitute the gray value *x*_*u*_ at point *u* for the variable *x* in Equation (3), the REF becomes as,


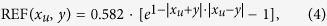


where *y* can be replaced by some proper rank statistics of the image block, such as min, max, mean, or median. Then, the function REF(*x*_*u*_, *y*) can be regarded as the belongingness of a pixel to an image block, or the measure of the difference between a pixel and its neighborhood.

### NLFI filtering

We make use of the falx cerebri to divide a brain MR image into the left and right parts. Subsequently, in each part (sub image), the object/background area is separated by a global threshold because global thresholding methods are easy to implement and also computationally less involved^23^, such as the Otsu, minimum error, and Parzen window estimate methods^24^. We adopt a global threshold that is chosen as,


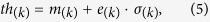


where *th*_(*k*)_ is the determined threshold in the *k-*th sub image (*k* = 1, 2), *m*_(*k*)_ and *σ*_(*k*)_ are the average and standard deviation of gray values of the *k-*th sub image, and *e*_(*k*)_ is a non-negative constant, respectively.

Each object/background area is processed by NLFI filtering (i.e., the fuzzification, hyperbolization and defuzzification operations are implemented on each object or background area in turn). Subsequently, a preparatory result is obtained via normalization. The chain of the NLFI is,


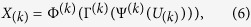


where *U*_(*k*)_ and *X*_(*k*)_ denote the input data and output data, and Ψ^(*k*)^, Γ^(*k*)^ and Φ^(*k*)^ denote the fuzzification, hyperbolization, and defuzzification operations.

Assume that the variable *y* in Equation (4) is the average gray value of object or background area in the *k-*th sub image, the fuzzification for the object area in the *k-*th sub image can be expressed as,


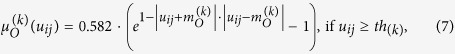


And the fuzzification for the background area is,


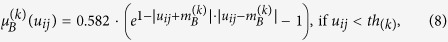


where *μ*_*O*_^(*k*)^ and *μ*_*B*_^(*k*)^ are the membership degrees of the object and background areas in the *k-*th sub image, *u*_*ij*_ is the gray value at point (*i*, *j*), *th*_(*k*)_ is a determined threshold according to Equation (5), and *m*_*O*_^(*k*)^ and *m*_*B*_^(*k*)^ are the average gray values of the object and background areas, respectively.

After the fuzzification, the sub images are converted into the membership planes. Then, an appropriate hyperbolization is necessary to enlarge the belonging of those points whose gray levels are close to the average gray value of the image block, and lessen the belonging of those points whose gray levels are far from that value. Therefore, for the membership degrees of the object area in the *k-*th sub membership plane, we adopt the following hyperbolization.


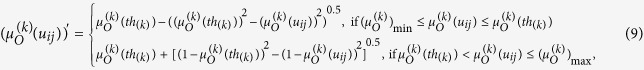


where 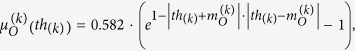


While the hyperbolization for the membership degrees of the background area is,


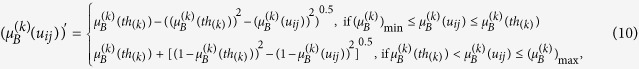


where 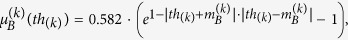 where (*μ*_*O*_^(*k*)^)’ and (*μ*_*B*_^(*k*)^)’ denote the hyperbolized membership degrees in the *k-*th sub block, (*μ*_*O*_^(*k*)^)_min_ and (*μ*_*O*_^(*k*)^)_max_ are the original minimum and maximum membership degrees of the object area, (*μ*_*B*_^(*k*)^)_min_ and (*μ*_*B*_^(*k*)^)_max_ are the original minimum and maximum membership degrees of the background area, respectively.

In order to improve the image quality, the hyperbolized membership plane needs to be inversely converted into the pixel plane. For the *k-*th sub hyperbolized membership plane, the defuzzification for the object area can be described as,


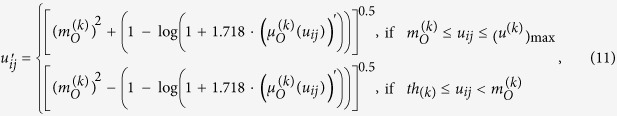


And the defuzzification for the background area is,


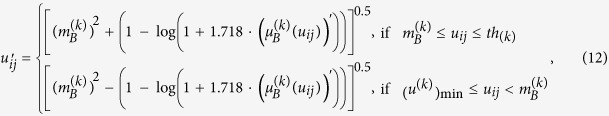


where *u*_*ij*_’ denotes the new pixel value at point (*i*,*j*), (*u*^(*k*)^)_min_ and (*u*^(*k*)^)_max_ denote the minimum and maximum gray values in the original *k-*th sub image.

Finally, a filtered brain tumor MR image can be obtained through the following normalization.


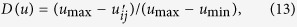


where *u*_*ij*_’ is the value at point (*i*, *j*) in the new pixel plane, and *u*_max_ and *u*_min_ denote the maximum and minimum values of the new pixel plane, respectively.

### AIFE scheme

The block diagram of AIFE scheme is shown in Fig. 3, intending to take information provided from different MR acquisitions, and enhance normal/abnormal structural regions of the brain while displaying the enhanced results as a single image. Therefore, an enhanced brain MR image can be obtained by,


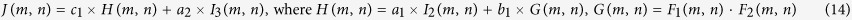


where *a*_1_, *a*_2_, *b*_1_, and *c*_1_ are the scaling factors, and +, × and · are the arithmetic addition, arithmetic multiplication and logical operations, respectively.

Nine groups of real patient data (comprising five primary-tumor and four cerebral-metastatic neoplasm patients) were used to test the proposed AIFE scheme, after the informed consent was obtained from all subjects. All experiments were carried out in accordance with guidelines and regulations provided and approved by the Institutional Review Board of Wuhan Institute of Physics and Mathematics (WIPM), Chinese Academy of Sciences (CAS).

## Results

### Metrics, Baseline Methods and Data

Mean opinion score (MOS) is a common index used in the subjective evaluation of image enhancement, aiming to ascertain which induces a pleasant impression in a human observer. In this test, several experts visually assess all original and processed brain tumor MR images. Every image is given a MOS value of 1–5, where a value of one means the worst visual quality and a value of five means the best quality.

Besides MOS, some objective measures are widely adopted to measure the performance of enhancement methods^25^,^26^, for example the contrast, EME (measure of enhancement), AME (Michelson law measure of enhancement), AMEE (Michelson law measure of enhancement by entropy), and SDME (second derivative-like measure of enhancement). The definition of contrast is independent of the actual range of gray levels in an image. All EME, AME, AMEE, and SDME measures divide an image into *k*_1_ × *k*_2_ blocks, and then calculate the average values of the measure results of all blocks in the whole image. The definitions of the contrast, EME, AME, AMEE, and SDME are displayed in Table 1, where *m* and *b* denote the average gray values of the foreground and background regions in an image. The size of the blocks could be composed of an odd number of pixels, such as, 3 × 3, 5 × 5 or 7 × 7. For each enhancement measure, a higher score denotes the better enhancement performance.

The AIFE scheme intends to improve the normal/abnormal brain tissues at the same time, such as focal edema, active tumor ingredients and anatomic structure of the brain. This scheme consists of the fuzzy filtering and fusion processes. Then, we select some widely-used fuzzy enhancement techniques as baseline methods for comparison, including the ZIM (Zadeh’s intensification)^27^, FHHM (fuzzy histogram hyperbolization)^16^, FRM (fuzzy relaxation)^28^, and *λ*-EM (*λ*-enhancement) methods^29^. In addition, a non-fuzzy enhancement approach, viz., the MFISTA (monotone version of fast iterative shrinkage/thresholding algorithm)^30^, is also chosen as the baseline method in this paper since this method is fast, simple and well studied for improving image quality.

The local ethics committee approved this study. Nine patients were involved in the study after they provided written informed consent, including five primary-tumor patients (mean age, 31 years; range, 18–47 years) as well as four cerebral -metastatic neoplasm patients (mean age, 50 years; range, 33–65 years). The primary-brain tumors were newly diagnosed. Details regarding the sex, age, tumor pathology, grade, and treatment for primary-tumor patients are listed in Table 2. Moreover, the details relating the sex, age, tumor metastasis origination, and treatment for cerebral-metastatic patients are also listed in Table 2. We discussed with several radiologists and surgeons, and they were unanimous that there was no grade for the cerebral-metastatic neoplasms.

Different sequences were utilized to produce MR images. All test images were randomly chosen from Hubei Cancer Hospital or Tongji Hospital, China. The images were scanned from December 2012 to March 2016. In this paper, we only utilized some axial T1-weighted, T2-weighted, contrast-enhanced T1-weighted, and T2-FLAIR images where the lesions can be seen, to compare the proposed method with baseline ones regarding enhancement performance. The reason is that our method aims to simultaneously improve the contrast of lesions and healthy tissues. However, the T2-weighted images were absent for the cerebral-metastatic neoplasm patients because such images were not provided by the hospital. We selected two images under each imaging modality in experiments. Then, the number of images for each primary-tumor patient was set to 8, while it was 6 for every cerebral-metastatic neoplasm patient. In addition, the window or leveling was fixed on all original and filtered MR images, and the fixed window/level was matched to original images.

### Enhancement Analysis

**Figure 1:**
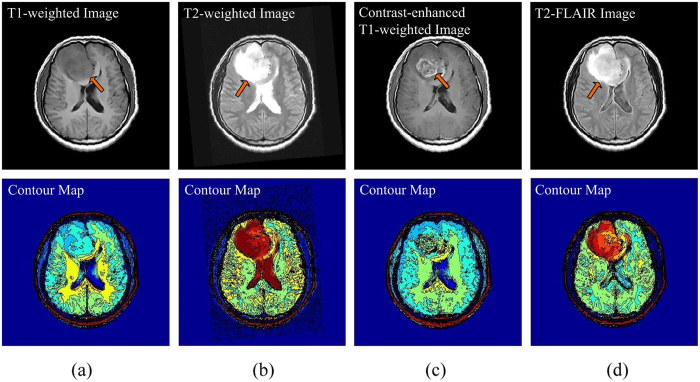
Representative brain tumor MR images under different imaging modes (upper) and the corresponding contour maps (lower). (**a**) T1-weighted image. (**b**) T2-weighted image. (**c**) Contrast-enhanced T1-weighted image. (**d**) T2-FLAIR image.

**Figure 2:**
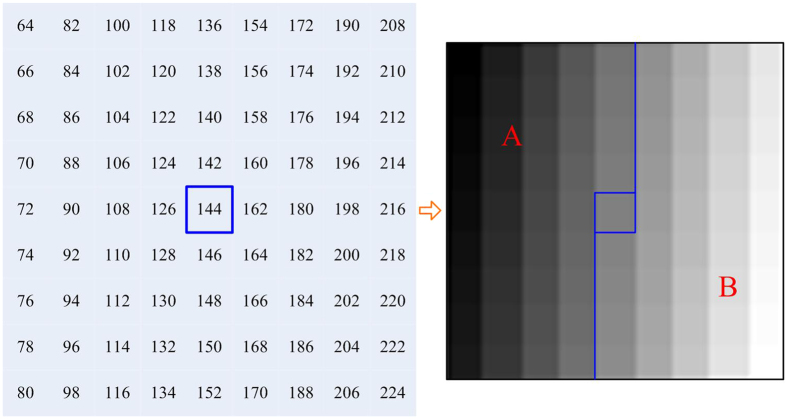
A local area with size of 9 × 9 (left) and the corresponding gray map (right). The map is separated into the part *A* and part *B* through the blue curve.

**Figure 3:**
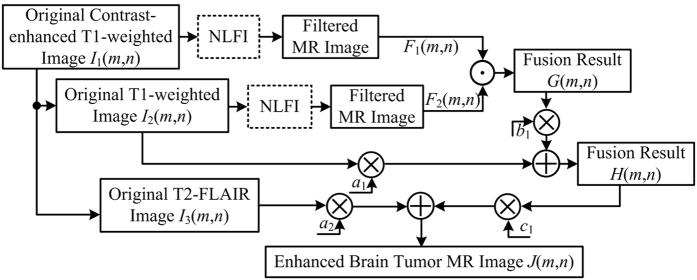
Block diagram of AIFE scheme, where *a*_1_, *a*_2_, *b*_1_, and *c*_1_ are the scaling factors.

**Figure 4:**
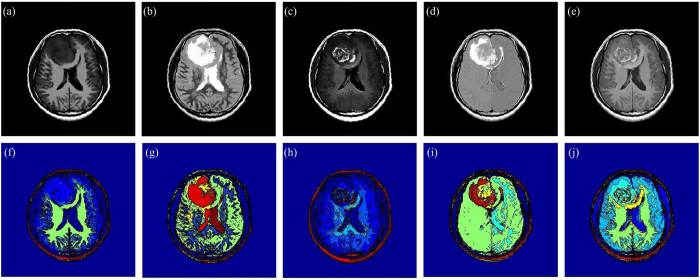
Enhancement analysis. (**a**–**d**) Filtered results of Fig. 1(a–d) obtained by using the NLFI filtering. **(e)** Enhanced result obtained by using the AIFE scheme. **(f**–**j**) Contour maps of (**a**–**e**).

For the diffuse astrocytoma MR images shown in Fig. 1(a–d), the filtered results acquired by using the NLFI filtering are shown in Fig. 4(a–d), and the contour maps are shown in Fig. 4(f–i), respectively. Figure 4(e) shows the enhanced result obtained by using the AIFE scheme, and the contour map is shown in Fig. 4(j), where the sum of parameters *a*_2_ and *c*_1_ in Equation (14) was set to 1.

Those filtered and enhanced results are evaluated by some radiologists and surgeons majoring in the tumor of head and neck and neurosurgery from the Hubei Cancer Hospital and Tongji Hospital. Compared with the original images, they agree that: i) In Fig. 4(a), the focal edema is more conspicuous, and the contrast between the focal edema and anatomic structure of the brain is clearer. Moreover, the homogeneity of white matter is better. ii) The contrast between the gray matter and white matter is enlarged in Fig. 4(b), and the focal edema is highlighted. Besides this, the mass effect (e.g., the midline and brain fold are pressed and pushed shift) is more obvious. iii) After the NLFI filtering, the active tumor ingredients are clearer, as shown in Fig. 4(c). The contrast between the active tumor ingredients and the normal brain tissue is evidently improved. iv) The influence of free water, the CSF and necrotic cystic are eliminated in Fig. 4(d), and the focal edema is more intact and clearer. v) Fig. 4(e,j) synchronously displays normal and abnormal brain tissues, such as, the brain fold, brain nuclei, corpus callosum, active tumor ingredients, and focal edema. This is helpful for surgeons and radiologists to discriminate and diagnose the focal edema and brain tumor, guide the operation, judge the prognosis, and plan the radiotherapy.

### Enhancement Measures

Baseline enhancement techniques are commonly utilized to improve the contrast and visual quality of MR images under diverse imaging modalities (e.g., T1-weighted, T2-weighted, contrast-enhanced T1-weighted, or T2-FLAIR brain MR images). The aim is consistent with that of the NLFI filtering process. Hence, sixty-four brain tumor MR images are processed through the ZIM, FRM, FHHM, *λ*-EM, MFISTA, NLFI, and AIFE methods. Including the original and processed images, there are 466 test MR images in total (5 × 4 × 2 × 7 + 4 × 3 × 2 × 7 + 2 × 9 = 466) for subjective and objective evaluation.

In the subjective test, seven experts assessed all original and processed MR images, including three radiologists and four surgeons majoring in the tumor of head and neck and neurosurgery. Each expert has at least ten years’ experience in the relevant field. All the observers were unaware of which algorithm was used in the non-processed and processed images, and all the test images were in a random order.

Table 3 lists the average MOS values of each observer, and the bottom row lists the ensemble average MOS values of the original and processed images categorized by the ZIM, FRM, FHHM, *λ*-EM, MFISTA, and AIFE algorithms. It can be seen from Table 3 that our method gives the best overall visual quality with scores of 4.76, 4.67, 4.43, 4.64, 4.29, 4.64, and 4.59, and the ensemble average value is 4.57. However, the FRM method obtains the worst quality with scores of 2.24, 1.14, 1.81, 1.42, 2.03, 2.15, and 2.25, and the ensemble average value is 1.86.

**Figure 5:**
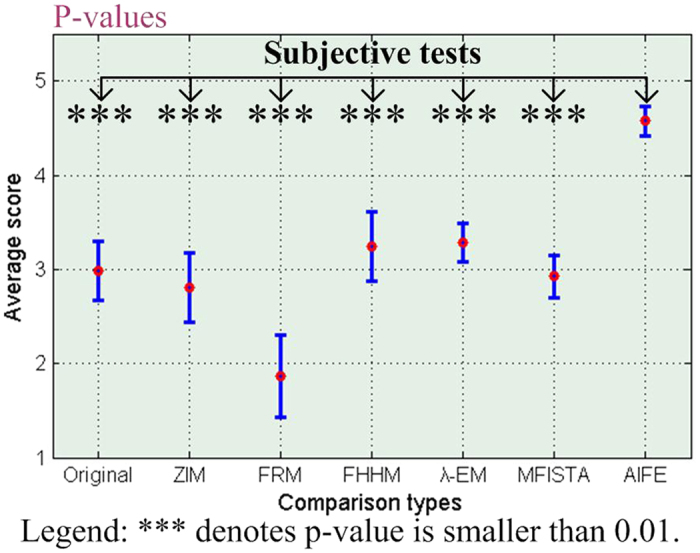
Statistical analysis of subjective evaluation scores.

Figure 5 shows the statistical analysis of subjective evaluation scores. The longitudinal ordinates denote MOS values, and the horizontal ordinates denote different enhancement methods. The p-values are the statistical difference between the scores of results obtained by using the AIFE and the scores of original images or results acquired by using the ZIM, FRM, FHHM, *λ*-EM, or MFISTA methods. The error bars denote the mean ± standard deviations of MOS values. Figure 5 indicates that all p-values in multiple comparisons are smaller than 0.01. Thus, we can draw a conclusion that the AIFE is statistically significantly different to the original as well as baseline methods in MOS comparison.

**Figure 6:**
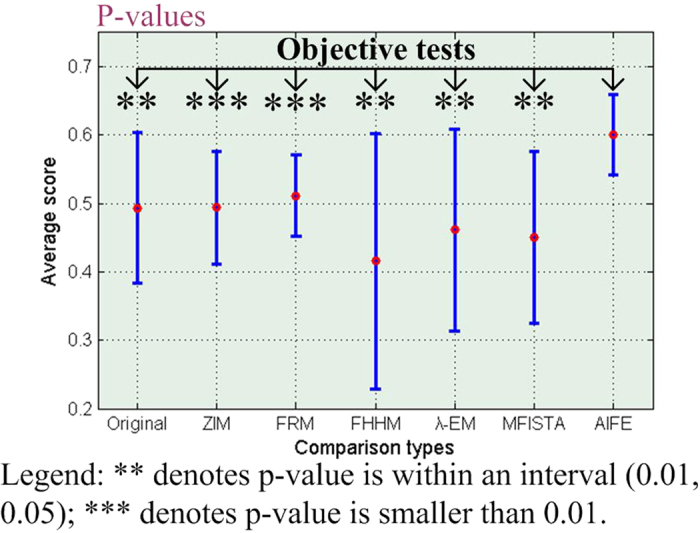
Statistical analysis of contrast values.

After that, we adopt the diverse enhancement measures to measure the visual quality of all 466 test MR images. Table 4 lists the average contrast values of the original and filtered images obtained by using the baseline and AIFE methods. These test images are categorized into nine groups according to patient types (as listed in Table 2). The measure results in Table 4 are the average contrast values of each group. For the calculation of contrast, some experts initially identify the true object (e.g., tumor or focal edema), then the foreground region surrounding the true object are determined. In Table 4, it can be seen that different algorithms produce various measure results for the nine groups of original and processed images. The ensemble average measures of nine groups categorized by the original, ZIM, FRM, FHHM, *λ*-EM, MFISTA, and AIFE methods are also listed in the bottom row of Table 4, that is, 0.4937, 0.4940, 0.5115, 0.4155, 0.4611, 0.4504, and 0.6003, respectively. The statistical analysis of the contrast is shown in Fig. 6, where the meaning of bar graphs is the same as that shown in Fig. 5. Table 4 suggests that the proposed method can achieve the best performance for all the nine groups of MR images. In addition, the significance test of contrast measures shown in Fig. 6 indicates that our method is significantly superior to the baseline methods.

Table 5 lists the ensemble average values of EME, AME, AMEE, and SDME measures for all test MR images. All the measure results indicate AIFE-enhanced MR images as the best. The conclusion derived from the contrast, EME, AME, AMEE, and SDME measure results is consistent with that from the subjective evaluation.

### Enhancement Performance

**Figure 7:**
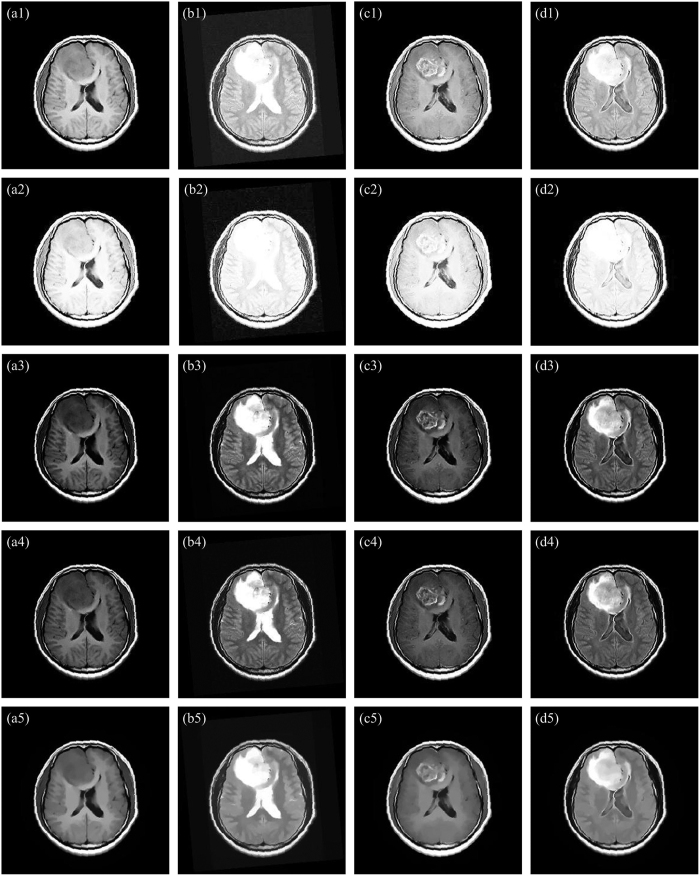
Filtered results of Fig. 1(a–d) obtained by using different baseline methods. (**a1**–**d1**), (**a2**–**d2**), (**a3**–**d3**), (**a4**–**d4**), and (**a5**–**d5**) Filtered results obtained by using the ZIM, FRM, FHHM, *λ*-EM, and MFISTA methods.

As for four brain tumor MR images shown in Fig. 1(a–d), the filtered results obtained by using the ZIM, FRM, FHHM, *λ*-EM, and MFISTA methods are shown in Fig. 7(a1–d1), (a2–d2), (a3–d3), (a4–d4), and (a5–d5), respectively. These filtered results are also evaluated by the radiologists and surgeons. They agree that: i) The contrast of the filtered results obtained by using the ZIM is inferior to that of original images, though the whole intensity of the images is enlarged. ii) Brain tissues, active tumor ingredients and focal edema are almost invisible in Fig. 7(a2–d2), which is disadvantageous in the discrimination and diagnosis of the focal edema and brain tumor. iii) The contrast of the filtered results obtained by using the FHHM and *λ*-EM is improved to some extent. iv) The fine structure is blurred in the filtered results acquired by using the MFISTA. v) Compared with the filtered results acquired by using the baseline methods, the contrast in Fig. 4(a–d) is more conspicuous, and the focal edema is clearer. vi) Brain fold, brain nuclei, corpus callosum, focal edema, and active tumor ingredients are not simultaneously displayed in the results obtained by using the baseline methods. In this way, each result cannot offset the deficiencies met by each MR imaging modality.

**Figure 8:**
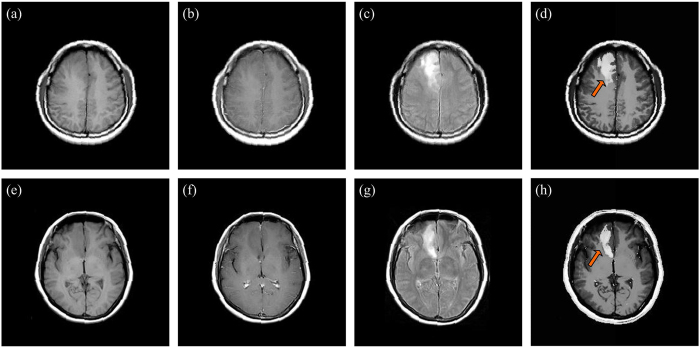
Original and enhanced images obtained by using our method. **(a**,**e**), **(b**,**f**), **(c**,**g**), and **(d**,**h)** Original T1-weighted, contrast-enhanced T1-weighted, T2-FLAIR, and enhanced results using the AIFE method.

There is a challenge in the contrast improvement of lesions which are usually characterized by T1/T2 sequences very similar to healthy tissues. Figure 8(a–c) display original T1-weighted, contrast-enhanced T1-weighted and T2-FLAIR MR images for the diffuse astrocytoma patient (viz., patient #1), and Fig. 8(e–g) show original MR images for the cerebral metastatic neoplasm from ovarian cancer (viz., patient #9). The enhanced results achieved by using our method is shown in Fig. 8(d,h), where the arrows denote the location of lesions (viz., focal edemas). It can be found that the gray features of lesions in T1-weighted and contrast-enhanced T1-weighted images are close to that of surrounding healthy tissues. This arouses the difficulty in distinguishing the normal/abnormal tissues of the brain, though the focal edemas are visible on T2-FLAIR images. However, through the proposed method, the brain fold, brain nuclei, corpus callosum, and focal edemas are synchronously highlighted, as shown in Fig. 8(d,h). This is helpful to discriminate and diagnose the focal edemas and brain tumor. Compared with original images, the contrast and the focal edemas in Fig. 8(d,h) are more conspicuous and clearer, and the white matter has better homogeneity.

**Figure 9:**
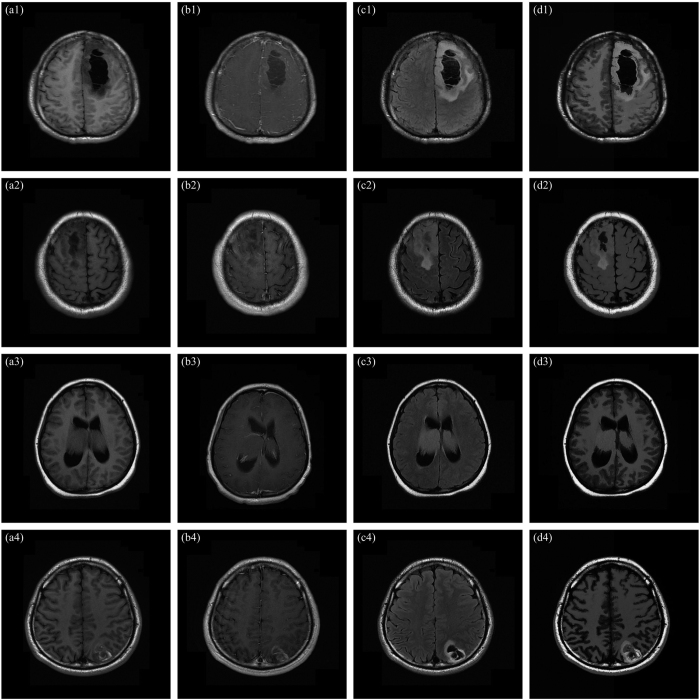
Original primary tumor images and improved results obtained by using our method. **(a1**–**a4)**, **(b1**–**b4)**, **(c1**–**c4)**, and (**d1**–**d4**) Original T1-weighted, contrast-enhanced T1-weighted, T2-FLAIR images, and improved results with the AIFE method.

In order to further compare conventional MR images with improved results obtained by using AIFE method, Fig. 9 shows original and improved images for primary-tumor patients (viz., patient #2, #3, #4, and #5). The tumor grades are low-grade. As well known by radiologists carrying out diagnosis based on MR images, different sequences (e.g., T1-weighted, T2-weighted, etc.) behave different and clearly defined contrasts. However, it can be seen from Fig. 9 that the contrasts in low-grade tumor images are low, especially in Fig. 9(b2). This arouses a great difficulty in judging a lesion. We can see that Fig. 9(d1–d4) have better contrast to noise ratio, in comparison to original MR images under different imaging modalities. In this way, the use of synchronous display could be advantageous in judging a lesion. Figure 9 suggests that our method is useful to have information from images acquired with different MR sequences fused in a single image.

To the best of our knowledge, conventional enhancement methods have not been exploited to synchronously display the diverse improved regions of interest of brain MR images under different imaging modalities in one image. However, this synchronous display is beneficial for radiologists and surgeons to understand and interpret abnormalities appeared in images. This is the aim of this paper. Therefore, the AIFE method is helpful to judge lesions by synchronously enhancing fine details in brain MR images with no *a priori* knowledge of the image contents.

## Discussion

In this study, a novel enhancement method (AIFE) was presented to enhance brain tumor MR images, which is good at highlighting fine details of brain MR images, and displays the improved abnormal/normal structural regions in a single image. This is helpful for radiologists and surgeons to understand the abnormalities that appear in a brain, and improve the disease diagnosis. The AIFE combines the intuitionistic fuzzy filtering (NLFI) with fusion operators. The membership function in the NLFI filtering can be achieved from other types of intuitionistic fuzzy generators, such as Sugeno’s or Yager’s generators. In addition, the NLFI system could be derived from other fuzzy filtering algorithms, e.g., logical FSs, type-I or type-II FSs, and it can be also designed as a combination of two or more different types of filters. This offers the AIFE scheme more robust characteristics.

Many coefficients are specified for the AIFE scheme, such as the threshold *e*_(*k*)_, *k* = 1,2, for each imaging mode *I*_*α*_(*m*, *n*), *α* = 1, 2, 3, and the scaling factors *a*_1_, *a*_2_, *b*_1_, *c*_1_ for the fusion. More coefficients provide the AIFE more robustness and design flexibility to suffice more complex and specific requirements in applications. Given some reasonable assumptions in specific applications, those coefficients could be represented by few variables. This will simplify the AIFE design and reduce the number of coefficients in practical applications. Moreover, the proposed AIFE scheme can be applied to multi-modality imaging systems like PET/MRI.

However, there are some limitations to our study. First, the number of patients in our study is small, although the total number of brain tumor MR images is relatively large compared with prior studies. Second, the AIFE only considers some axial MR images, without combining coronary and sagittal images, which cannot hold a comprehensive understanding of brain tumor characteristics, such as tumor size, morphology, and margin. In this case, it is difficult to distinguish between benign and malignant lesions, prognosticate tumor grades, and differentiate between primary and metastatic tumors, etc. During the clinical practice, different types of tumors could receive different treatments, for example primary tumors and cerebral metastatic neoplasms (as displayed in Table 2). Accordingly, the differentiation of between primary tumors and cerebral metastatic neoplasms is the key point for our future work. Third, the AIFE requires contrast-enhanced T1- weighted images that are mainly influenced by vascular leakage of the contrast agent at sites where the blood-brain barrier is disrupted. In certain cases, patients who are treated with anti-angiogenic agents have their tumor vasculature sealed off and there is a decrease in contrast enhancement, which may not reflect true tumor regression on post-therapy MR images. As a result, the usefulness of this technique will be limited in patients receiving this type of treatment. Also, the effectiveness of the AIFE scheme should be further explored (especially for the first stage gliomas), although the proposed method is helpful to diagnose low-grade tumors (as shown in Figs 4 and 9) to a certain extent. As well known, low-grade tumors (such as the first stage gliomas) are painful to be wholly resected, insensitive to radiotherapy and chemotherapy, and easy to relapse. Furthermore, such types of tumors are usually characterized by T1 and T2 sequences very similar to healthy brain tissues. Hence, the improvement of low-grade tumors is difficult and challenging. But this illustrates prospects in future study. Last, the use of synchronous presentation (integration of T1-weighted, contrast- enhanced T1-weighted and T2-FLAIR images) potentially introduces confusion in understanding lesions. Although the contrast is improved and the focus is highlighted in AIFE results, some detailed anatomical information is possibly lost, for example sulci and gyri. If there is a small metastatic foci elsewhere, this fine structure is potentially masked in AIFE results. We know that the structure of brain tumor is complicated, due to the coexistence of edema, necrosis, ischemia, inflammation, and hyperplasia of small blood vessels. If the difference of such structure is highlighted, the potential of the AIFE will be further strengthened.

## Conclusion

This paper proposes a new IFSs-based scheme (AIFE) for enhancing brain tumor MR images, aiming to simultaneously improve the visual quality of normal/abnormal structural regions of a brain and display the results in an image. The AIFE divides an input image into sub images, successively followed by different intuitionistic fuzzification, hyperbolization and defuzzification operations implemented on the respective object and background areas in each sub image, and finally achieves a highlighted result through nonlinear fusion operators. The proposed scheme provides more robustness and flexibility, in order to satisfy more specific and complex requirements in clinical applications. In comparison to baseline methods such as the ZIM, FRM, FHHM, *λ*-EM, and MFISTA methods, the AIFE has better performance with respect to the improvement of the contrast and visual quality of abnormalities in brain MR images (such as the focal edema and/or active tumor ingredients), and making these abnormalities more discriminable. Moreover, the AIFE synchronously shows the brain fold, focal edema, brain nuclei, corpus callosum, and/or active tumor ingredients in an image, which is helpful to understand and interpret lesions. Experimental results demonstrate that the AIFE scheme has great potential for understanding and determining the normal and abnormal functional regions of the brain, and improving disease detection and diagnosis.

## Additional Information

**How to cite this article**: Deng, H. *et al*. Adaptive Intuitionistic Fuzzy Enhancement of Brain Tumor MR Images. *Sci. Rep.* **6**, 35760; doi: 10.1038/srep35760 (2016).

**Publisher’s note:** Springer Nature remains neutral with regard to jurisdictional claims in published maps and institutional affiliations.

## Figures and Tables

**Table 1:** Definitions of several enhancement measures.

Name	Definition
Contrast	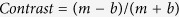
EME	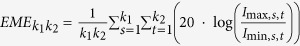
AME	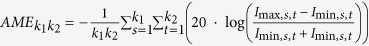
AMEE	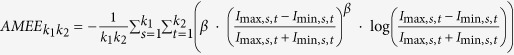
SDME	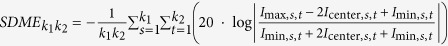

Where the image *I* is divided into *k*_1 _× *k*_2_ blocks, *β* is a constant, *I*_max,*s*,*t*_ and *I*_min,*s*,*t*_ are the maximum and minimum gray values of the pixels in each block, and *I*_center,*s*,*t*_ is the gray value of the center pixel in each block, respectively.

**Table 2:** Details of the primary-tumor and cerebral-metastatic neoplasm patients.

Patient	Sex	Age	Tumor pathology	Grade	Treatment
**Primary tumor**					
#1	male	30	diffuse astrocytoma	II	surgery + radiotherapy
#2	male	44	glioma	II-III	surgery + radiotherapy
#3	male	47	temporal lobe tumor	II	surgery + radiotherapy
#4	female	18	thalamic glioma	II	surgery + radiotherapy
#5	male	18	diffuse astrocytoma	II	surgery + radiotherapy
**Patient**	**Sex**	**Age**	**Metastasis origination**	**Treatment**	
**Cerebral metastatic neoplasm**					
#6	male	58	laryngeal carcinoma	one cycle chemotherapy with PF regimen	
#7	female	33	infiltrating ductal carcinoma of breast	three cycles chemotherapy with CEF regimen	
#8	male	65	adenocarcinoma	resection of right frontal lobe + radiotherapy	
#9	female	43	ovarian cancer	resection of anterior cranial fossa + radiotherapy	

Where the PF regimen denotes the cisplatin combined 5-fluorouracil regimen, and the CEF regimen is the cyclophosphamide, epirubicin and 5-fluorouracil regimen. The tumor grade is according to the WHO (World Health Organization) classification.

**Table 3:** Subjective comparison of original and processed brain tumor MR images obtained by using different algorithms.

Expert	Original	ZIM	FRM	FHHM	*λ*-EM	MFISTA	AIFE
#1	2.68	2.54	2.24	2.62	3.01	2.69	4.76
#2	2.42	2.17	1.14	2.84	3.07	2.52	4.67
#3	3.12	3.01	1.81	3.41	3.40	2.99	4.43
#4	3.15	2.69	1.42	3.38	3.26	3.01	4.64
#5	3.29	3.12	2.03	3.40	3.24	3.14	4.29
#6	3.14	3.25	2.15	3.66	3.62	3.09	4.64
#7	3.10	2.85	2.25	3.37	3.39	3.03	4.59
Average	2.99	2.80	1.86	3.24	3.28	2.92	4.57

Note: 1 = Bad, 2 = Poor, 3 = Fair, 4 = Good, 5 = Excellent.

**Table 4:** Comparison of contrast values for the original and processed images obtained by using different methods.

Patient	Original	ZIM	FRM	FHHM	*λ*-EM	MFISTA	AIFE
#1	0.5681	0.5402	0.5328	0.5655	0.5711	0.5185	0.6528
#2	0.5792	0.5558	0.5587	0.5669	0.5780	0.5149	0.6778
#3	0.3315	0.4162	0.4255	0.1492	0.2405	0.2839	0.5351
#4	0.5103	0.4653	0.5426	0.4446	0.5228	0.5161	0.5395
#5	0.3390	0.3463	0.4053	0.1413	0.2464	0.2608	0.5428
#6	0.5810	0.5719	0.5566	0.5633	0.5735	0.5512	0.6224
#7	0.5778	0.5676	0.5482	0.5608	0.5712	0.5616	0.6582
#8	0.5782	0.5566	0.5563	0.5161	0.5259	0.5371	0.6282
#9	0.3786	0.4259	0.4774	0.2317	0.3208	0.3091	0.5457
Average	0.4937	0.4940	0.5115	0.4155	0.4611	0.4504	0.6003

**Table 5:** Comparison of EME, AME, AMEE, and SDME measures based on different algorithms.

	Original	ZIM	FRM	FHHM	*λ*-EM	MFISTA	AIFE
EME	20.2749	20.7695	22.9403	23.3479	20.8594	20.8926	24.1259
AME	13.2903	15.0042	18.1989	9.4970	12.6578	10.2109	23.2258
AMEE	0.0590	0.0765	0.0686	0.0527	0.0536	0.0619	0.0983
SDME	27.4103	29.3389	31.9228	21.4244	26.2005	27.8934	36.4746
